# Association between sarcopenia and cognitive impairment in community-dwelling population

**DOI:** 10.1097/CM9.0000000000001310

**Published:** 2020-12-07

**Authors:** Hui Zhu, Heng-Dong Li, Bei-Li Feng, Lu Zhang, Zai-Xing Zheng, Yue Zhang, Dong-Juan Wang, Zi Xiong, Jun-Fei Kang, Jia-Chang Jin, Jie Lin, Hong-Hua Ye

**Affiliations:** 1 Department of Experimental Medical Science, HwaMei Hospital, University of Chinese Academy of Sciences, Ningbo, Zhejiang 315010, China; 2 Key Laboratory of Diagnosis and Treatment of Digestive System Tumors of Zhejiang Province, Ningbo, Zhejiang 315010, China; 3 Department of Cardiology, HwaMei Hospital, University of Chinese Academy of Sciences, Ningbo, Zhejiang 315010, China.

Cognitive impairment is a neurodegenerative change, involving the progression from mild cognitive impairment to dementia.^[1]^ Sarcopenia is often considered geriatric and is characterized by a progressive and generalized decrease in skeletal muscle mass and strength.^[2]^ Interestingly, several common pathogenic mechanisms are involved in both sarcopenia and cognitive disorders. The risk factors for sarcopenia include mitochondrial dysfunction, oxidative stress, chronic inflammation, and hormonal changes, all of which are potential causes of cognitive impairment.^[3]^ Consequently, cognitive impairment may be closely related to sarcopenia. This study aims to examine the relationship between cognitive impairment and sarcopenia among community-dwelling elderly adults.

A cross-sectional survey on health and chronic disease management was conducted from November 2016 to February 2017 in the Ximen community of Ningbo, China. All included subjects were aged 65 years or older and independent in their daily lives. Subjects with serious diseases, including severe cardiac, cerebrovascular, pulmonary, musculoskeletal diseases, neurologic disorders, or cancers, were excluded. Finally, 923 subjects were recruited for the study, after excluding 124 participants who had missing data in their physical or cognitive function assessments.

This study was conducted in accordance with the *Declaration of Helsinki* and was approved by the Ethics Committee of HwaMei Hospital, University of Chinese Academy of Sciences (No. PJ-NBEY-KY-2016-020-01). Written informed consent was obtained from all subjects during their epidemiological interview, and subject anonymity has been preserved.

Demographic characteristics and anthropometric measurements were collected by trained staff. Sarcopenia was diagnosed based on low muscle mass, low muscle strength, low physical performance, or all of these, according to the Asian Working Group for Sarcopenia. Low muscle mass was defined as a skeletal muscle mass index (skeletal muscle mass/height^2^) of <7.0 kg/m^2^ in men and <5.7 kg/m^2^ in women. Low muscle strength was identified as a handgrip strength measurement of <26 kg in men and <18 kg in women. Low physical performance was determined as a 4-meter gait speed of <0.8 m/s. The mini-mental state examination (MMSE) is a general tool for evaluating participants’ cognitive function. The total MMSE score ranged from 0 to 30, and cognitive impairment was defined as a score of <20 for illiterate participants, <22 for participants who had undergone education for 1 to 6 years, or <27 for participants with more than 6 years of education.

Participants were classified into two groups: cognitive impairment and normal. Data analysis was performed using Stata 13 (Stata Corporation, College Station, TX, USA). Univariate logistic regression models and propensity score matching were performed to assess the association between cognitive impairment and sarcopenia. The propensity score was constructed using multivariable logistic regression, with cognitive impairment as the binomial dependent variable and all the observed covariates related to cognitive impairment as predictor variables. A two-tailed *P* value < 0.05 was considered statistically significant.

There were 823 participants (89.2%) in the normal group and 100 participants (10.8%) in the cognitive impairment group [Supplementary Table 1]. Participants with cognitive impairment were older than those without (74.58 ± 5.51 *vs.* 72.10 ± 5.13 years, *P* < 0.001). Significant differences in education levels were also identified (*P* = 0.009). In addition, the cognitive impairment group had significantly lower handgrip strength (cognitive impairment group: 23.31 ± 8.33 kg, normal group: 25.24 ± 8.39 kg, *P* < 0.001) and 4-meter gait speed (cognitive impairment group: 1.07 ± 0.28 m/s, normal group: 1.19 ± 0.24 m/s, *P* < 0.001) than the normal group. A comparison of variables between groups after propensity score matching is presented in Supplementary Table 2.

The results of the univariate logistic regression analyses showed a significant association with cognitive impairment for both low physical performance (odd ratio [OR]: 2.69, 95% confidence interval [CI]: 1.42–5.08) and sarcopenia (OR: 2.04, 95% CI: 1.16–3.57). However, the statistical significance was lost after controlling potential covariates through propensity score matching [Table 1].

**Table 1:** Univariate logistic regression model and propensity score matching model to determine the association between sarcopenia and cognitive impairment.

	Model 1		Model 2	
Parameters	Cruded OR (95% CI)	*P*	Adjusted OR (95% CI)	*P*
Age (years)				
65–69	Reference		Reference	
70–74	1.32 (0.74–2.35)	0.348	1.15 (0.53–2.50)	0.716
75–79	3.21 (1.86–5.52)	<0.001	1.44 (0.70–2.97)	0.321
80–84	2.40 (1.18–4.89)	0.016	0.88 (0.36–2.15)	0.784
85+	3.37 (0.68–16.67)	0.137	2.31 (0.2–26.94)	0.505
BMI (kg/m^2^)				
18.5–24	Reference		Reference	
<18.5	1.04 (0.30–3.61)	0.953	0.60 (0.14–2.67)	0.502
24–28	0.91 (0.58–1.43)	0.693	1.11 (0.6–2.03)	0.746
>28	0.72 (0.36–1.44)	0.357	0.85 (0.34–2.09)	0.718
Gender	0.93 (0.61–1.41)	0.729	1.27 (0.73–2.22)	0.395
Education				
Illiteracy	Reference		Reference	
≤6 years of education	1.00 (0.22–4.55)	0.996	1.16 (0.19–6.98)	0.870
>6 years of education	2.20 (0.51–9.39)	0.288	2.50 (0.44–14.09)	0.299
Live alone	0.85 (0.47–1.54)	0.585	0.85 (0.39–1.86)	0.692
Drinking	0.97 (0.64–1.48)	0.897	0.85 (0.48–1.49)	0.568
Smoking	1.09 (0.68–1.75)	0.730	0.71 (0.39–1.31)	0.279
Hypertension	1.07 (0.700–1.63)	0.749	0.81 (0.46–1.43)	0.473
Diabetes	0.91 (0.52–1.61)	0.756	1.08 (0.51–2.27)	0.849
Dyslipidemia	1.06 (0.65–1.72)	0.822	1.17 (0.62–2.21)	0.627
BFR				
Q1 (<27.76)	Reference		Reference	
Q2 (27.76–32.76)	1.17 (0.63–2.16)	0.627	1.19 (0.53–2.65)	0.673
Q3 (37.76–37.52)	1.49 (0.82–2.68)	0.189	1.33 (0.61–2.87)	0.473
Q4 (>37.52)	1.22 (0.66–2.25)	0.525	1.47 (0.65–3.34)	0.352
Low muscle mass	1.43 (0.91–2.24)	0.120	1.05 (0.58–1.90)	0.879
Low handgrip strength	1.54 (1.00–2.39)	0.052	1.31 (0.73–2.37)	0.367
Low physical performance	2.69 (1.42–5.08)	0.002	1.65 (0.68–4.00)	0.271
Sarcopenia	2.04 (1.16–3.57)	0.013	2.22 (0.94–5.21)	0.067

BFR has been computed on the basis of the formula, body fat mass (kg)/weight (kg)^∗^100%. Model 1 shows a univariate logistic regression model. Model 2 shows propensity score-matched analyses based on age, gender, BMI, drinking, smoking, hypertension, diabetes, dyslipidemia, and BFR. OR: Odds ratio; CI: Confidence interval; BMI: Body mass index; BFR: Body fat rate; Q: Quartile.

Previous cross-sectional and longitudinal studies have been conducted to determine the relationship between sarcopenia and cognitive status, which have yielded inconsistent conclusions.^[4]^ Our findings revealed no association between sarcopenia as previously defined and cognitive impairment, after adjusting for potential covariates. The results of the present study were consistent with available evidence from a French observational, prospective, multicenter cohort study of community-dwelling elderly women.^[5]^ Although there appear to be common underlying etiological mechanisms between the two, the present analyses did not support a relationship between sarcopenia and cognitive impairment.

Several limitations of the present study should be noted. First, the cross-sectional design of the study could not explain the causal inferences for cognitive function. Second, our results showed an association between sarcopenia and cognitive impairment in the elderly population; however, the findings may not apply to other populations. Finally, subjective cognitive impairment was examined using the MMSE. However, the results achieved through another psychological test such as the neurobehavioral cognitive status examination may differ from ours.

Regardless of these limitations, the findings of our study, based on an elderly community population in China, may provide some evidence for the pathogenesis of cognitive function. Moreover, we adjusted for potential covariates, including age, sex, education, body fat rate, drinking, smoking, hypertension, diabetes mellitus, and dyslipidemia, to decrease the rate of false positives.

In conclusion, our findings indicated no significant association between sarcopenia and cognitive impairment in a community-dwelling population. Further prospective cohort studies with different populations, including sarcopenic patients or participants with dementia, are necessary to confirm these findings.

## Funding

This work was supported by the Ningbo Medical Science and Technology Project (No. 2016Z01) and the Zhejiang Provincial Public Service and Application Research Foundation (No. LGF20H250001).

## Conflicts of interest

None.

## Supplementary Material

Supplemental Digital Content
