# Supplementary material for: Association between sarcopenia and cognitive impairment in community-dwelling population
Source: Chin Med J (Engl). 2020 Dec 7;134(6):725–7. doi: 10.1097/CM9.0000000000001310 (PMC7989998; doi:10.1097/CM9.0000000000001310)
Supplement: Supplemental Digital Content [file cm9-134-725-s001.pdf]

Supplementary Table 1. Descriptive characteristics of study participants (*n*=923)

Characteristics	Total subjects (*n* = 923)	Cognitive normal group (*n* = 823)	Cognitive impairment group (*n* = 100)	*P*
Age (years)	72.37 ± 5.23	72.1 ± 5.13	74.58 ± 5.51	<0.001
65–69	376 (40.74)	350 (42.53)	26 (26)	<0.001
70–74	269 (29.14)	245 (29.77)	24 (24)	
75–79	182 (19.72)	147 (17.86)	35 (35)	
80–84	86 (9.32)	73 (8.87)	13 (13)	
85+	10 (1.08)	8 (0.97)	2 (2)	
Gender				
Male	382 (41.39)	339 (41.19)	43 (43)	0.729
Female	541 (58.61)	484 (58.81)	57 (57)	

Education				
Illiteracy	31(3.36)	29 (3.52)	2 (2)	0.009
≤6 years of education	278 (30.12)	260 (31.59)	18 (18)	
>6 years of education	608 (65.87)	528 (64.16)	80 (80)	
BMI (kg/m^2)	24.73 ± 3.35	24.75 ± 3.34	24.53 ± 3.51	0.535
18.5–24	379 (41.06)	335 (40.7)	44 (44)	0.826
<18.5	25 (2.71)	22 (2.67)	3 (3)	
24–28	392 (42.47)	350 (42.53)	42 (42)	
>28	127 (13.76)	116 (14.09)	11 (11)	
Live alone				
No	146 (15.82)	132 (16.04)	14 (14)	0.585
Yes	773 (83.75)	687 (83.48)	86 (86)	
Drinker				

No	539 (58.40)	480 (58.32)	59 (59)	0.897
Yes	384 (41.60)	343 (41.68)	41 (41)	
Smoker (%)				
No	696 (75.41)	622 (75.58)	74 (74)	0.729
Yes	227 (24.59)	201 (24.42)	26 (26)	
Hypertension				
No	420 (45.50)	376 (45.69)	44 (44)	0.749
Yes	503 (54.50)	447 (54.31)	56 (56)	
Dyslipidemia				
No	230 (24.92)	206 (25.03)	24 (24)	0.822
Yes	693 (75.08)	617 (74.97)	76 (76)	
Diabetes				
No	138 (14.95)	122 (14.82)	16 (16)	0.755

Yes	785 (85.05)	701(85.18)	84 (84)	
BFR	32.56 ± 7.08	32.53 ± 7.1	32.79 ± 7.01	0.736
Q1 (<27.76)	231 (25.03)	210 (25.52)	21 (21)	0.610
Q2 (27.76–32.76)	230 (24.92)	206 (25.03)	24 (24)	
Q3 (37.76–37.52)	232 (25.14)	202 (24.54)	30 (30)	
Q4 (>37.52)	230 (24.92)	205 (24.91)	25 (25)	
Cognitive domains				
Orientation	9.88 ± 0.55	9.93 ± 0.3	9.48 ± 1.38	<0.001
Memory	2.92 ± 0.31	2.96 ± 0.21	2.59 ± 0.65	<0.001
Attention and calculations	4.13 ± 1.34	4.37 ± 1.12	2.14 ± 1.33	<0.001
Recall capacity	2.42 ± 0.88	2.54 ± 0.78	1.43 ± 1.04	<0.001
Language ability	8.75 ± 0.61	8.82 ± 0.49	8.2 ± 1.03	<0.001
MMSE score	28.1 ± 2.43	28.61 ± 1.79	23.84 ± 2.82	<0.001

Sarcopenia domains				
Grip strength (kg)	25.03 ± 8.40	25.24 ± 8.39	23.31 ± 8.33	0.030
Four-meters gait speed (m/s)	1.17 ± 0.27	1.19 ± 0.24	1.07 ± 0.28	<0.001
Skeletal muscle index (kg/m^2)	6.68 ± 0.97	6.68 ± 0.96	6.62 ± 1.02	0.555
Low handgrip				
No	667 (72.26)	603 (73.27)	64 (64)	0.051
Yes	256 (27.74)	220 (26.73)	36 (36)	
Low speed				
No	862 (93.39)	776 (94.29)	86 (86)	0.002
Yes	61 (6.61)	47 (5.71)	14 (14)	
Low muscle				

No	687 (74.43)	619 (75.21)	68 (68)	0.118
Yes	236 (25.57)	204 (24.79)	32 (32)	
Sarcopenia				
No	825 (89.38)	743 (90.28)	82 (82)	0.011
Yes	98 (10.62)	80 (9.72)	18 (18)	

Data are presented as *n* (%) or mean ± standard deviation .BMI: Body mass index; BFR: Body fat rate; MMSE:Mini-mental state examination; kg: Kilograms; m/s:Meter per second; kg/m^2:Kilogram per meter square; Q: Quartile.BFR has been computed on the basis of the formula, Body fat mass (kg)/Weight (kg)*100%.

Supplementary Table 2. Comparison of variables between cognitive impairment and cognitive normal groups after propensity score matching

Characteristics	Cognitive normal group (*n* = 100)	Cognitive impairment group (*n* = 100)	*P*
Age (years)	74.33 ± 5.69	74.58 ± 5.51	0.748
65–69	30 (30)	26 (26)	0.751
70–74	24 (24)	24 (24)	
75–79	28 (28)	35 (35)	
80–84	17 (17)	13 (13)	
85+	1 (1)	2 (2)	
Gender			
Male	49 (49)	43 (43)	0.478

Female	51 (51)	57 (57)	
Education			
Illiteracy	4 (4)	2 (2)	0.053
≤6 years of education	31(31)	18 (18)	
>6 years of education	64 (64)	80 (80)	
BMI (kg/m^2)	24.44 ± 3.35	24.53 ± 3.51	0.850
18.5–24	44 (44)	44 (44)	0.833
<18.5	5 (5)	3 (3)	
24–28	38 (38)	42 (42)	
>28	13 (13)	11 (11)	
Live alone			
No	84 (84)	86 (86)	0.692
Yes	16 (16)	14 (14)	

Drinker			
No	55 (55)	59 (59)	0.568
Yes	45 (45)	41 (41)	
Smoker			
No	67 (67)	74 (74)	0.278
Yes	33 (33)	26 (26)	
Hypertension			
No	39 (39)	44 (44)	0.473
Yes	61 (61)	56 (56)	
Dyslipidemia			
No	27 (27)	24 (24)	0.626
Yes	73 (73)	76 (76)	
Diabetes			

No	17 (17)	16 (16)	0.849
Yes	83 (83)	84 (84)	
BFR	32.59 ± 6.84	32.79 ± 7.01	0.844
<27.76	26 (26)	21 (21)	0.809
27.76–32.76	25 (25)	24 (24)	
37.76–37.52	28 (28)	30 (30)	
>37.52	2 1(21)	25 (25)	
Cognitive domains			
Orientation	9.88 ± 0.38	9.48 ± 1.38	0.006
Memory	2.97 ± 0.17	2.59 ± 0.65	<0.001
Attention and calculations	4.3 ± 1.16	2.14 ± 1.33	<0.001
Recall capacity	2.5 ± 0.73	1.43 ± 1.04	<0.001
Language ability	8.83 ± 0.4	8.2 ± 1.03	<0.001

MMSE score	28.48 ± 1.79	23.84 ± 2.82	<0.001
Sarcopenia domains			
Grip strength (kg)	25.13 ± 8.42	23.31 ± 8.33	0.126
Four-meters gait speed (m/s)	1.12 ± 0.24	1.07 ± 0.28	0.191
Skeletal muscle index (kg/m^2)	6.61 ± 0.9	6.62 ± 1.02	0.936
Low handgrip			
No	70 (70)	64 (64)	0.367
Yes	30 (30)	36 (36)	
Low speed			
No	91 (91)	86 (86)	0.268
Yes	9 (9)	14 (14)	
Low muscle			
No	69 (69)	68 (68)	0.879

Yes	31 (31)	32 (32)	
Sarcopenia			
No	91 (91)	82 (82)	0.063
Yes	9 (9)	18 (18)	

Data are presented as *n* (%) or mean ± standard deviation .BMI: Body mass index; BFR: Body fat rate; MMSE:Mini-mental state examination; kg: Kilograms; m/s:Meter per second; kg/m^2:Kilogram per meter square; Q: Quartile.BFR has been computed on the basis of the formula, Body fat mass (kg)/Weight (kg)*100%.
